# Intelligent Diagnostic Prediction and Classification Models for Detection of Kidney Disease

**DOI:** 10.3390/healthcare10020371

**Published:** 2022-02-14

**Authors:** Ramesh Chandra Poonia, Mukesh Kumar Gupta, Ibrahim Abunadi, Amani Abdulrahman Albraikan, Fahd N. Al-Wesabi, Manar Ahmed Hamza, Tulasi B

**Affiliations:** 1 Department of Computer Science, CHRIST (Deemed to be University), Bangalore 560029, India; rameshcpoonia@gmail.com (R.C.P.); tulasi.b@christuniversity.in (T.B.); 2 Department of Computer Science & Engineering, Swami Keshvanand Institute of Technology, Management & Gramothan (SKIT), Jaipur 302017, India; mukeshgupta@skit.ac.in; 3 Department of Information Systems, Prince Sultan University, P.O. Box No. 66833 Rafha Street, Riyadh 11586, Saudi Arabia; i.abunadi@psu.edu.sa; 4 Department of Computer Sciences, College of Computer and Information Sciences, Princess Nourah bint Abdulrahman University, P.O. Box 84428, Riyadh 11671, Saudi Arabia; aalbraikan@pnu.edu.sa; 5 Department of Computer Science, College of Science & Art at Mahayil, King Khalid University, Abha 61421, Saudi Arabia; 6 Department of Computer and Self Development, Preparatory Year Deanship, Prince Sattam bin Abdulaziz University, Al-Kharj 16273, Saudi Arabia; mahamza@psau.edu.sa

**Keywords:** usability score artificial intelligence, medical information systems, image matching, machine learning algorithms, morphological operations

## Abstract

Kidney disease is a major public health concern that has only recently emerged. Toxins are removed from the body by the kidneys through urine. In the early stages of the condition, the patient has no problems, but recovery is difficult in the later stages. Doctors must be able to recognize this condition early in order to save the lives of their patients. To detect this illness early on, researchers have used a variety of methods. Prediction analysis based on machine learning has been shown to be more accurate than other methodologies. This research can help us to better understand global disparities in kidney disease, as well as what we can do to address them and coordinate our efforts to achieve global kidney health equity. This study provides an excellent feature-based prediction model for detecting kidney disease. Various machine learning algorithms, including k-nearest neighbors algorithm (KNN), artificial neural networks (ANN), support vector machines (SVM), naive bayes (NB), and others, as well as Re-cursive Feature Elimination (RFE) and Chi-Square test feature-selection techniques, were used to build and analyze various prediction models on a publicly available dataset of healthy and kidney disease patients. The studies found that a logistic regression-based prediction model with optimal features chosen using the Chi-Square technique had the highest accuracy of 98.75 percent. White Blood Cell Count (Wbcc), Blood Glucose Random (bgr), Blood Urea (Bu), Serum Creatinine (Sc), Packed Cell Volume (Pcv), Albumin (Al), Hemoglobin (Hemo), Age, Sugar (Su), Hypertension (Htn), Diabetes Mellitus (Dm), and Blood Pressure (Bp) are examples of these traits.

## 1. Introduction

Kidney disease affects over 750 million people worldwide, a figure that is growing. Kidney disease is a condition that affects people all over the world, but the disease’s prevalence, identification, and treatment are all very different. Renal failure is the leading cause of death among people living in modern society. Cigarette smoking, excessive alcohol consumption, high cholesterol, and a variety of other risk factors all play a role in the disease. The kidney is a vital organ in the human body, performing a variety of vital functions. Despite the fact that kidney disease is better understood in developed countries, new research indicates that the condition is more prevalent in developing countries. The primary function is to collect waste and excess fluid from the circulatory system and excrete it via the kidneys via urine. If the function of this organ is compromised, the amount of harmful liquids and wastes in our systems may have disastrous consequences [1]. It is critical to emphasize that there are two kinds of kidney disease: acute kidney disease and chronic (long-term) kidney disease [2]. The most common type of kidney illness is acute renal disease. Chronic kidney failure is characterized by a progressive decline in kidney function over time (usually years). When the kidney’s blood supply is cut off, the flow of urine is hampered by an enlarged prostate, or the kidney itself is injured and becomes ineffective, this type of kidney failure occurs. As a result of a chronic renal condition, kidney failure does not occur overnight. In the early stages of the disease, the patient exhibits no signs or symptoms of the illness. Patients who have had diabetes and high blood pressure for a long time are more likely to develop this syndrome. Patients who have been exposed for an extended period of time to lead-based medications and poisons are at risk of developing this disease. According to a poll, this condition affects a large number of people in our country, and thousands of people die from it each year. Only the most affluent countries have access to renal failure treatment. According to the World Health Organization, only 11% of the world’s population receive adequate treatment for renal failure. Because they cannot afford dialysis or a kidney transplant, low-income patients die of renal failure. Patients who are identified and treated early on have a better chance of avoiding renal failure entirely. Scientists have developed a number of methods for detecting kidney disease at an early stage [3,4]. Patients’ doctors may inform them ahead of time. Taking preventative measures before things get out of hand is a viable option.

### Chronic Kidney Disease

Humans have two kidneys that are roughly the size of a fist. Their primary purpose is to filter blood. They remove waste and excess water, which turn into urine. They also help to keep the body’s chemical balance, control blood pressure, and produce hormones. Chronic kidney disease means that the kidneys are damaged and are unable to filter blood as effectively as they should. This damage can cause waste to accumulate in the body and cause other issues that can be harmful to health. The most common causes of chronic kidney disease are diabetes and high blood pressure. Kidney damage occurs gradually over a long period of time. Many people have no symptoms until their kidney disease is advanced. Only blood and urine tests can inform you if you have kidney disease. Treatments cannot cure kidney disease, but they can help to slow its progression. They include blood pressure medications, blood sugar control medications, and cholesterol-lowering medications. Chronic kidney disease can worsen over time. It can occasionally result in kidney failure. Dialysis or a kidney transplant will be required if your kidneys fail. Based on population studies from developed countries, a systematic review found a mean prevalence of 7.2% in individuals older than 30 years. According to WHO data, it affects approximately 10% of the adult population and more than 20% of those over the age of 60, and it is undoubtedly underdiagnosed. The prevalence of CKD can reach 35–40% in patients followed up in primary care for diseases as common as high blood pressure (HBP) or diabetes mellitus (DM). The magnitude of the problem is magnified by the increase in morbidity and mortality, particularly cardiovascular mortality, caused by renal deterioration. CKD is thought to be the common final destination of a group of pathologies that affect the kidney in a chronic and irreversible manner. Once the diagnostic and therapeutic options for primary kidney disease have been exhausted, CKD necessitates common protocols of action that are, in general, independent of it. The most common causes of ACKD are described below, along with links to further information. More than one cause frequently coexists and worsens kidney damage.

In this work, the primary objective is to identify the best early-stage prediction model [5] for renal disease based on the most optimal attributes possible [6]. The following sub-goals are included:

- Review the existing approaches for the detection of kidney disease.
- Determine the best feature by applying various feature selection techniques.
- Build various prediction models on a kidney dataset using different machine learning algorithms and analyze their accuracy in the detection of kidney disease.

The rest of the article is organized as follows: Section 2 provides a review of the literature on the detection of kidney disease. Section 3 proposes a method for detecting kidney disease that makes use of machine learning and feature extraction. Section 4 discusses the kidney dataset, experimental results, and comparisons with existing methods. Section 5 discusses the conclusion and future work.

## 2. Related Works

The diagnosis of kidney illness using machine learning algorithms is an emerging subject of computer vision in healthcare. Because of their great accuracy in identifying illnesses, these procedures are gaining prominence. Using machine learning algorithms, such as decision trees, J48, Support Vector Machine (SVM), and others, researchers have developed several methods for identifying kidney illness. This section describes previous research ideas proposed by a variety of scholars.

Boukenze, B. et al. [6] suggested a machine learning-based method for identifying renal disorders. They employed the k-nearest neighbors algorithm (KNN), support vector machine (SVM), decision tree, and artificial neural network (ANN) machine learning algorithms. They used a number of performance measures to evaluate the accuracy of prediction models. They observed that the decision tree-based model outperformed all other models in diagnosing chronic failure, with an accuracy of 63 percent.

A. Salekin and colleagues employed SVM, KNN, and random forest techniques to build prediction models. They based their findings on a dataset of 400 cases. There were 24 properties in each record. Different machine learning algorithm-based models produced variable degrees of accuracy, it was revealed. The accuracy of the decision tree-based model was 98 percent, which was greater than that of earlier models.

H. Polat et al. [7] predicted renal disease using the SVM machine learning technique. They had a 97.5 percent accuracy rate. In order to enhance the accuracy, they applied a variety of feature selection methodologies. They improved the accuracy by 1% by employing feature selection.

Panwong, P. et al. [8] proposed an approach using KNN, NB, and decision tree classifiers. They also reduced the number of features by using the wrapper technique. Using the decision tree technique, they attained a maximum accuracy of 85 percent.

Dulhare, U. N. et al. [9] suggested a technique for diagnosing kidney illness using the naive Bayes machine learning algorithm in combination with the R attribute selector. They were 97.5% accurate in diagnosing renal illness.

Vasquez-Morales et al. [10] developed a neural network classifier based on massive quantities of CKD data, and the model proved to be 95 percent accurate in its predictions. To predict the advancement of diabetic kidney disease, Makino et al. [11] collected patient diagnoses and treatment information from textual data in an attempt to predict the progression of diabetic kidney disease.

According to Ren et al. [12], they developed a prediction model for diagnosing chronic kidney disease (CKD) using data from electronic health records (EHR). Based on a neural network architecture, the proposed model encoded and decoded textual and numerical data from electronic health records (EHR). A deep neural network model for identifying chronic renal disease was developed by Ma F. et al. [13]. Comparing the supplied model with ANN and SVM, the accuracy of the given model was the highest.

Almansour and colleagues [14] utilized machine learning to develop a technique for preventing chronic kidney disease. Researchers used machine learning classification methods, such as SVM and ANN, to make their findings. The experiments revealed that ANN outperformed SVM in terms of accuracy, with a 99.75% accuracy rate.

J. Qin and colleagues [15] presented a machine learning strategy for diagnosing chronic kidney disease (CKD) in its early stages. In order to construct their models, they used logistic regression, random forest, SVM, naive Bayes classifier, KNN, and the feedforward neural network as techniques. With an accuracy rating of 99.75%, the random forest classification model was shown to be the most accurate.

Z. Segal and colleagues [16] developed an ensemble tree-based machine learning algorithm (XGBoost) for the diagnosis of kidney disease in its early stages. Models such as random forest, CatBoost, and regression with regularization were used to compare the results of the stated model. All matrices were improved by using the proposed model, which had c-statistics of 0.93, sensitivity of 0.715%, and specificity of 0.958, among other improvements.

Khamparia et al. [17] developed a deep learning model for the early identification of chronic kidney disease (CKD) that employed a stacked autoencoder model to extract features from multimedia data and was published in Nature Communications. The authors used a SoftMax classifier to predict the final class, which they found to be accurate. Using the UC Irvine Machine Learning Repository (UCI) chronic kidney disease (CKD) dataset [18], it was revealed that the recommended model outperformed standard classification algorithms when compared to the data set in question.

Ebiaredoh Mienye Sarah A. et al. [19] developed a robust model for predicting chronic kidney disease (CKD) by combining an enhanced sparse autoencoder (SAE) with Softmax regression. The autoencoders in our proposed model achieved sparsity by penalizing the weights, as previously stated. Because the SoftMax regression model was specifically tailored for the classification task, the proposed model performed wonderfully in the testing environment. On the chronic kidney disease (CKD) data set, the proposed model had a precision of 98 percent, according to the researchers. When it came to performance, the proposed model outperformed other already available strategies.

According to Zhiyong Pang et al. [20], a fully automated computer-aided diagnostic approach that employed breast magnetic resonance imaging to differentiate between malignant and benign masses was proposed.

Using a combination of the support vector machine and the ReliefF feature selection approaches, the texture features were selected for use. It was found that this method was 92.3% accurate.

Chen, G. et al. [21] developed a model for identifying Hepatitis C virus infection that used the Fisher discriminating analysis method with an SVM classifier to obtain a more accurate diagnosis. The comparison of the proposed methodology to current methods showed that the hybrid method outperformed all other methods, reaching the highest classification accuracy of 96.77%. The authors of this paper developed a breast cancer diagnosis model [22]. Artificial neural networks are used to classify breast cancer based on qualities that have been selected using sequential forward and backward selection processes. SBSP obtained the highest level of accuracy, with a score of 98.75%.

Table 1 outlines prior studies by different researchers. According to the table, researchers employed multiple machine learning algorithm-based prediction models to predict renal disorders. The accuracy of these models varied and was inadequate. We noticed that many researchers did not pre-process their data and used no feature selection strategy.

## 3. Support Vector Machine

The first concepts and foundational principles of SVM were provided by the statistical learning theory (structural risk minimization). It can be used in classification and nonlinear regression. This broad classification of SVM can be further subdivided into two subcategories: linear SVM (linear SVM) and nonlinear SVM (nonlinear SVM) [28].

L-SVM [29] training data of different types are classified using linear SVM, which classifies training data by giving Class 1 to the “+1” and Class 2 to the “−1” symbols, then uses the mathematical notation

(1) $$\left\{{\left\{x_{i},y_{i}\right\}}_{i-1}^{T},x_{i}\in R^{m},y_{i}\in \left\{-1,+1\right\}\right\}\;w\cdot x+b=0$$

here *w* is the weight vector, *x* is the input dataset, and *b* is a bias in the hyper plane, which is referred to as a displacement. Bias is used to make sure that the hyper plane [11] is positioned correctly following movement in a horizontal plane. Thus, prejudice is affected by training with bias. A hyper plane has its parameters, which are *w* and *b*. A decision surface G. Chen et al. (2020) [29] is considered to be a function when SVM is used for classification.

(2) f(x)=sign(w⋅x+b)

SVM generally serves to increase the marginal distance of the data set and therefore enhance the distinguishing function, allowing better categorization. Improving the hyperplane’s distinguishing function is a quantic programming issue.

(3) $$minimize\;L_{p}=\frac{1}{2}∥w{∥}^{2}\;subject\;to\;\;y_{i}\left(x_{i}\cdot w+b\right)-1\geq 0,\;i=1,\ldots ,l\;$$

To solve the initial minimization issue, we apply the Lagrange theory:

(4) $$\begin{array}{cc} & L_{D}\left(\alpha \right)\;=\;\overset{l}{\underset{i=1}{\sum }}\alpha_{i}-\frac{1}{2}\overset{l}{\underset{i=1}{\sum }}\overset{l}{\underset{j=1}{\sum }}\alpha_{i}\alpha_{j}y_{i}y_{j\left(x_{i}x_{j}\right)}\\ \mathrm{subject}\;\mathrm{to} & \overset{l}{\underset{i=1}{\sum }}\alpha_{i}y_{i}=0,i=1,\ldots ,l\\ & \alpha_{i}\geq 0,i=1,\ldots ,l \end{array}\;$$

In the end, the linear divisive decision-making function has been completed.

(5) $$f\left(x\right)\;=\;\mathrm{sign}\left(\overset{n}{\underset{i=1}{\sum }}y_{i}\alpha_{i}^{*}\left(x\cdot x_{i}\right)+b^{*}\right)$$

To sum up, when *f*(*x*) > 0, it indicates that the sample is marked +1 and is in the same category as samples marked with “+1”; otherwise, it indicates that the sample is marked −1 and is in the same category as samples marked with “−1”. Linear hyper planes [30] cannot properly identify data points when training data include noise. Slack variables *ξ_i_* are introduced to the constraint, resulting in a modification of the original (3):

(6) $$\begin{array}{cc} \mathrm{minimize} & \frac{1}{2}∥w{∥}^{2}+C\left(\overset{l}{\underset{i=1}{\sum }}\xi_{i}\right)\\ \mathrm{subject}\;\mathrm{to} & y_{i}\left(x_{i}\cdot w+b\right)-1+\xi_{i}\geq 0,\;i=1,\ldots ,l\\ & \xi_{i}\geq 0,\;i=1,\ldots ,l \end{array}$$

The position of the border and the classification point are separated by a distance of *ξ_i_*; in this case, *C* represents the cost of the training data classification mistake, as specified by the user. A lower *C* value means that the margin will be narrower, suggesting that fault tolerance has a lower chance of working in the event of a problem [31,32]. The fault tolerance rate will be larger if *C* is lower. The linear inseparable issue (also known as the infinitely large linear problem) will degenerate into a linear separable problem as *C*→∞. In this instance, the parameters and the optimal solution of the target function may be found by using the Lagrangian coefficient [33,34] in order to solve the linear inseparable dual optimization issue; hence, the solution of the linear inseparable dual optimization problem is as follows:

(7) $$\begin{array}{cc} \mathrm{Max} & L_{D}\left(\alpha \right)\;=\;\overset{l}{\underset{i=1}{\sum }}\alpha_{i}-\frac{1}{2}\overset{l}{\underset{i=1}{\sum }}\overset{l}{\underset{j=1}{\sum }}\alpha_{i}\alpha_{j}y_{i}y_{j}\left(x_{i}x_{j}\right)\\ \mathrm{subject}\;\mathrm{to} & \overset{l}{\underset{i=1}{\sum }}\alpha_{i}y_{i}=0,i=1,\ldots ,l\;\\ & 0\leq \alpha_{i}\leq C,i=1,\ldots ,l \end{array}\;$$

Finally, the linear decision-making function is

(8) $$f\left(x\right)\;=\;\mathrm{sign}\left(\overset{n}{\underset{i=1}{\sum }}y_{i}\alpha_{i}^{*}\left(x\cdot x_{i}\right)+b^{*}\right),$$

a support vector machine whose operation can include nonlinear inputs (nonlinear SVM). In the case where we cannot separate training samples using linear SVM, we may apply feature transformation, such as the function φ, to convert original 2-D data into a new, high-dimensional feature space that allows us to solve linear separable problems. SVM can use the kernel technique to effectively conduct nonlinear classification utilizing an approach known as the kernel trick. For the time being, there are many diverse foundational components being put forward. Differentiating distinct data characteristics with respect to different core functions allows for more efficient computation with SVMs [7]. Of the very common fundamental functions, these four functions have something in common:

Linear kernel function:

(9) $$K\left(x_{i},y_{i}\right)=x_{i}^{t}\cdot y_{j}$$

Polynomial kernel function:

(10) $$K\left(x_{i},y_{j}\right)={\left(\gamma x_{i}^{t}x_{j}+r\right)}^{m},\;\gamma >0$$

Radial basis kernel function:

(11) $$K\left(x_{i},y_{j}\right)=exp\left(\frac{-∥x_{i}-y_{j}{∥}^{2}}{2\sigma^{2}}\right),\;\gamma >0$$

Sigmoid kernel function:

(12) $$K\left(x_{i},y_{j}\right)=tanh\left(\gamma x_{i}^{t}\cdot y_{j}+r\right)$$

This study utilizes the emissive core function, because settings such as *γ* and *C* can increase computation efficiency and lower SVM complexity.

## 4. Materials and Methods

**Figure 1:**
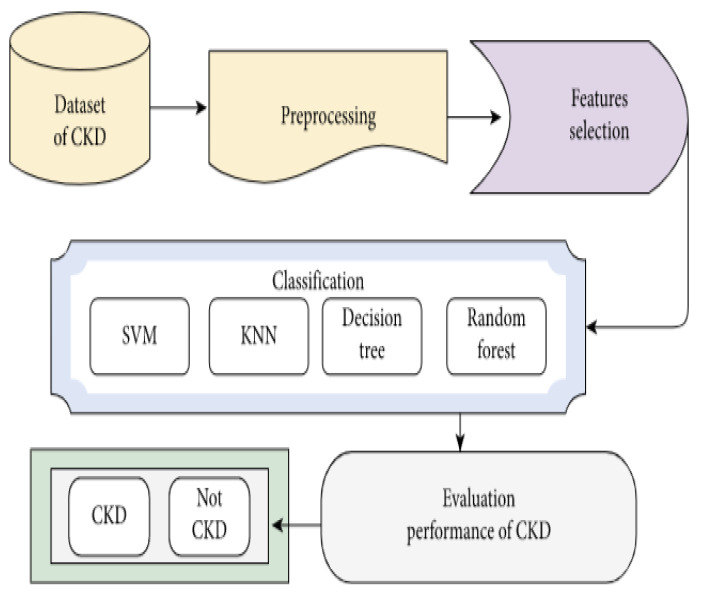
Detection of chronic kidney disease using recursive feature elimination and classification algorithms. CKD: Chronic Kidney Disease; SVM: Support Vector Machine; KNN: K-Nearest Neighbors.

The proposed strategy is based on data mining framework as shown in Figure 1. Data mining employs computational approaches at the intersection of artificial intelligence, machine learning, statistics, and database systems [35]. Data mining is predicated on the idea that data can be analyzed from a variety of perspectives. The “Knowledge Discovery in Databases” (KDD) process is employed in this study to extract unknown patterns from web data [36]. This section describes the suggested method for detecting kidney disease. The availability of kidney disease care is directly affected by each country’s public policies and financial situation. A lower dialysis–to–transplant ratio, for example, suggests that more affluent countries have a higher rate of kidney transplantation.

### 4.1. Kidney Disease Dataset

In this work, we used a dataset of 400 patients, each with 24 attributes [18,37]. The dataset had 250 records of patients who were suffering from kidney disease and 150 medical records for completely healthy people. This dataset has medical data for different age groups. It has 50 records of people less than 30 years old and 55 records of people greater than 70 years old. The remaining records belong to people aged 31–69. From the various studies, it was found that people of any age group may suffer from kidney disease. Therefore, there is no risk of bias in evaluating the performance of prediction models. Table 2 shows the details of the various kidney disease-related attributes.

**Figure 2:**
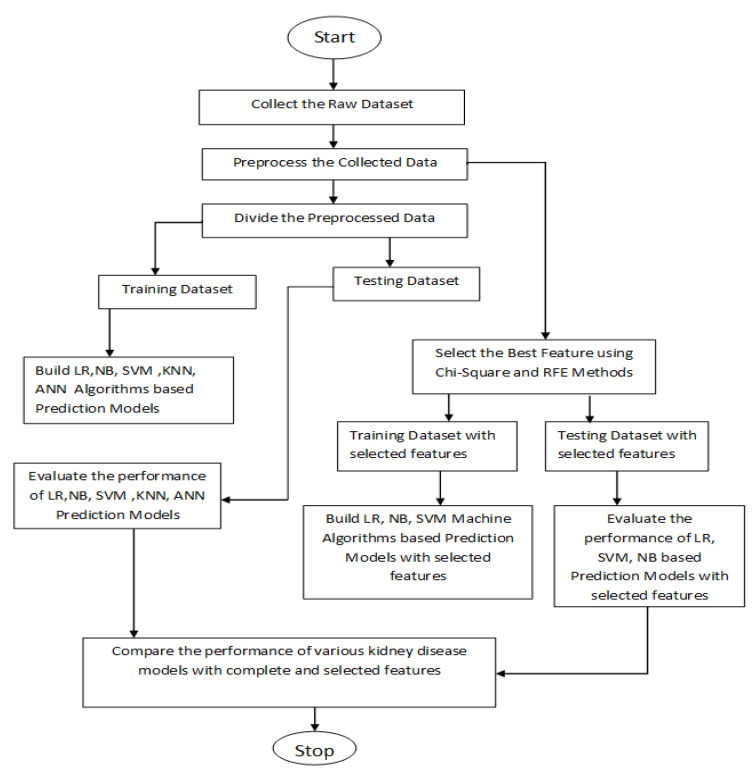
Flow chart of the proposed model. LR: Logistic Regression; NB: Naïve Bayes; SVM: Support Vector Machine; KNN: Nearest Neighbors; ANN: Artificial Neural Network; RFE: Recursive Feature Elimination.

To explain the proposed approach in an easy and efficient manner, a flow chart of the whole procedure is given in Figure 2 and the steps are explained one–by–one as follows:

### 4.2. Proposed Algorithm

Procedure: The proposed approach for the detection of kidney disease

Input: Dataset of kidney disease records

Output: Performance of the prediction models in detecting kidney disease.

It has the following steps:

Step 1: The Glomerular Filtration Rate (GFR) is the most often utilized measure of kidney health function in CKD medical therapy. In order to calculate which, the formula uses information such as the patient’s blood creatinine, age, race, gender, and other variables. As is widely accepted, the standard formula for renal disease modification of diet (MDRD).

(13) $$GfR=186\times {\left(create\;\right)}^{-1.154}\times {\left(age\;\right)}^{-0.203}\times \left(\frac{\frac{\mathrm{mL}}{\min}}{173\;m^{2}}\right)$$

Then preprocess the collected data: In this step, we preprocess the collected kidney disease dataset. In the original dataset, the ‘rbc’ and ‘pc’ columns have normal, abnormal, and empty values. The ‘rbc’ and ‘pc’ columns have 150 and 120 entries without any values, respectively. In this dataset, the ‘pcc’ and ‘ba’ columns have ‘present’ and ‘not present’ values. The ‘cad’, ‘pe’, ‘htn’, ‘dm’, and ’ane’ columns have the values ‘yes’ and ‘no’. Also, in this dataset, ‘appet’ has the values ‘poor’ and ‘good’. Therefore, preprocessing of this dataset is a mandatory task for correct results. In this step, the empty values are replaced by NaN. We converted nominal values to binary values as follows:

1. In the ‘rbc’ and ‘pc’ columns, ‘normal’ and ‘abnormal’ nominal values are replaced with 1 and 0, respectively.
2. In the ‘pcc’ and ‘ba’ columns, the ‘present’ and ‘nonpresent’ values are replaced with 1 and 0, respectively.
3. In the ‘htn’, ‘pe’, ’ane’, ‘dm’, and ‘cad’ columns, the values ‘yes’ and ‘no’ are replaced with 1 and 0, respectively.
4. Finally, in the ‘appet’ column, ‘good’ and ‘poor’ are replaced with 1 and 0, respectively.

In the next step, null values are replaced by the average value of that particular column’s values.

Step 2. Observe the relationship between different features. In this step, we find the relationship between input and target features. We found that ‘pot’ and ‘ba’ are weakly related to the target feature.

Step 3. Divide the dataset. In this step, we divide the dataset into training and testing datasets using an 80:20 ratio. It means that 80% of data are used for training and 20% of data are used for testing purposes.

Step 4. Set the parameters of the machine learning algorithms. In this step, the kidney disease dataset’s processed features are used with machine learning algorithms to build prediction models. We used Logistic Regression, Naive Bayes, Support Vector Machine, K-Nearest Neighbors (KNN), and Artificial Neural Network (ANN) machine learning algorithms. We applied a 10-fold cross validation for building the prediction models.

Let ϕ(x) be a ridge basis function, nonconstant, limited, and monotonically growing. If K is a compact subset on $R^{n}$, and $f\left(x_{1},\ldots ,x_{n}\right)$ is a real-valued continuous function on *K*, then *K* may be represented as a subset of *R*^*n*, where *f* is a collection of real numbers. Given an arbitrary positive parameter, there are integer N and real parameters

(14) $$v_{j},\theta_{j},w_{ij}\;\mathrm{for}\;i=1,\ldots ,n\;\mathrm{and}\;j=1,\ldots ,m.\;f^{\sim }\left(x_{1},\ldots ,x_{n}\right)=\sum_{j=1}^{m}v_{j}\phi_{j}\left(\sum_{i=1}^{n}w_{ij}x_{i}+\theta_{j}\right)+d$$

it satisfies the condition

(15) $$max_{X\in K}\left|f^{\sim }\left(X\right)-f\left(X\right)\right|<\varepsilon$$

We are saying that, for every given *ε* > 0, there exists a three-layer network, where the hidden layer represented by the ridge basis function *ϕ*(*x*) and whose input–output function is *f*˜(*x*_1_, …, *x_n_*), which has a $max_{X\in K}\left|f^{\sim }\left(X\right)-f\left(X\right)\right|<\varepsilon$ mapping function *f*˜(*x*_1_, …, *x_n_*) that results in *f*(*x*_1_, …, *x_n_*) being greater than or equal to *ε*.

Step 5. Feature selection. In this step, we select the best features using the Recursive Feature Selection (RFE) and Chi-Square feature selection methods. As our kidney disease data set was a labeled dataset, we used the wrapper and filter technique that is the supervised feature selection technique. As we discussed earlier, the supervised feature selection techniques were divided into three categories, which had different methods in each category.

For feature selection, we used S=(U,C∪D) and B⊆C, where *S* is the set of attributes of feature and attribute set D with respect to the conditional attribute subset B, then the evaluation function for feature selection is defined by

(16) $$\sigma \left(B,D\right)=\frac{1}{N}\left(\sigma_{B}\left(D_{1}\right)+\sigma_{B}\left(D_{2}\right)+\cdots +\sigma_{B}\left(D_{N}\right)\right)$$

In this case, *N* is the number of decision classes generated by the decision attribute set *D*, and is equal to $\sigma_{B}\left(D_{i}\right),i=1,2,\ldots ,N$, reflecting the uncertainty measure of each decision class, and σ(B,D) describes the integrated uncertainty degree of blocks $D_{1},D_{2},\ldots ,D_{N}$.

Recursive Feature Elimination (RFE) is a feature selection algorithm of the wrapper type. Internally, it employs filter-based techniques that are distinct from the filter approach. It has two important configuration options: a. it specifies the number of features to be selected, and b. it specifies the machine learning algorithm used in feature selection. In the first case, it searches for a subset of features by considering all of the features in the training dataset and removing them until the required number of features remains. In the second case, it employs a machine learning algorithm that ranks features [38] based on their importance. It removes the least important features and then repeats the model fitting process. The process is repeated until the specified number of features remain.

The Chi-Squared feature selection method investigates the relationship between the input features and the target class. In this test, the Chi-Square value is calculated for each input feature and the target class. It has the required number of features, as well as the highest Chi-Square scores. We used the formula below to calculate the chi-square metric (Xc2) between each target class feature and each input feature. It chooses only the input features with the highest Chi-Squared values.

Chi-Square feature selection in data with *m* attribute values and *k* class labels as output. Then, the value of $\chi^{2}$ is

(17) $$\chi^{2}=\overset{m}{\underset{i=1}{\sum }}\overset{k}{\underset{j=1}{\sum }}\frac{{\left(O_{ij}-E_{ij}\right)}^{2}}{E_{ij}}$$

where$\;O_{ij}$ is the observed frequency.

Step 6. Build the prediction model using the selected features. In this step, again, we applied 10-fold cross validation with the selected features and various machine learning algorithms to build different prediction models.

Step 7. Finally, the performance of prediction models with all features and selected features are compared.

## 5. Results and Analysis

To assess the performance of machine learning approaches, researchers use a variety of performance metrics. To evaluate and compare the performance of proposed prediction models, we used the precision, recall, F-measure, and accuracy performance measures.

### 5.1. Performance Measures

Accuracy is calculated by dividing the number of test records by the number of successfully classified records. The percentage of True Positive (TP) records to the total number of True Positive (TP) records in a certain class is called precision. There are two types of recall: true positives and false negatives. The total number of records properly categorized to the total number of records in a class is known as the recall ratio (FN). The precision, recall, F-measure, and accuracy were calculated using the following formulas:

(18) $$Precision_{i}=\frac{TP_{i}}{TP_{i}+FP_{i}}\;\;\;\;$$

(19) $$Recall_{i}=\frac{TP_{i}}{TP_{i}+FN_{i}}\;\;\;\;\;\;\;\;\;\;\;\;\;\;$$

(20) $$F_{\beta }=\frac{\left(1+\beta^{2}\right)\;\;precision*recall}{\beta^{2}*precision+recall}\;$$

where *β* is a parameter that can be used to give the importance to any one precision or recall.

Accuracy is commonly used as a measure for categorization techniques.

(21) $$Accuracy_{i}=\frac{TP_{i}+TN_{i}}{TP_{i}+FP_{i}+FN_{i}+TN_{i}}$$

where *TP_i_* is the number of records correctly classified as belonging to the kidney disease class, *FP_i_* is the number of records incorrectly classified as having kidney disease, *FN_i_* is the number of records that were not classified as having a kidney disease, and *TN_i_* is the number of images that were not assigned to the correct kidney disease class.

Precision (*P*) is a metric that quantifies the proportion of correct positive outcomes among all possible outcomes. It is computed as follows:

(22) P=TP/(TP+FP) 

Specificity: The system’s ability to accurately recognize the absence of impurities in the ghee picture is measured in this category. To obtain it, the number of true negatives recognized in the photographs must be counted and divided by the amount of pure milk included in the images. It was utilized to determine the specificity of the data.

(23) Specificity (SP)=(TN)/(TN+FP) 

Mean: Means are a straightforward approach commonly used in pure mathematics, as well as in analysis and computing; a wide variety of means have been invented to perform these duties. During an image processing competition, the technique of filtering by the mean is evaluated as abstraction filtering and is utilized for noise reduction.

(24) $$X^{-}=\frac{\sum_{i=0}^{n}X_{i}}{n}$$

A measure of variability or diversity in statistics, the standard deviation is the most widely used measure available to researchers. In the context of image processing, it indicates what fraction of variance or dispersion occurs between the predicted value and the observed value. An extremely low standard deviation suggests that the data points have a strong tendency to be extremely near to one another. A large standard deviation, on the other hand, shows that the data points are evenly distributed throughout a wide range of values.

(25) $${X^{-}}_{rms}=\sqrt{\frac{\sum_{X_{i=1}}^{n}{\left(X_{i}-X^{-}\right)}^{2}}{\left(n-1\right)}}$$

We used Anaconda, an enterprise-ready, secure, and scalable data science platform, and Spyder to build and analyze the prediction approaches (Python 3.6). To evaluate the proposed method’s performance, we downloaded a kidney disease dataset containing 400 patient records. We pre-processed the data to remove null values and for other purposes. The data set was divided into two parts: training and testing, with 80 percent of the records in training and 20% in testing. Using machine learning algorithms, such as Logistic Regression, NB, SVM, K-Nearest Neighbors (KNN), and Artificial Neural Network, we developed a variety of prediction models (ANN).

The data correlation matrix was represented using Heatmap [6]. It shows how different features interact with one another. It is a useful visualization technique for comparing the values of any two features. A positive correlation indicates that, as the value of a feature increases, so does the value of the target variable. It could be negative, implying that increasing the value of a feature decreases the value of the target variable. The heatmap was created with the help of the seaborn library. It visually displays which features are closely related to the target variable. By simply looking at the different color tones used, it can be determined which value is higher, lower, and so on. A heatmap correlation matrix of kidney disease data was displayed. It showed that the Ane, Bgr, Bu, Sc, Pcv, Al, Hemo, Age, Su, Htn, Dm, and Bp characteristics were highly related to the target variable (represented in green color). This means that raising these parameter values raises the risk of kidney disease.

### 5.2. Prediction Models with All Features

**Figure 3:**
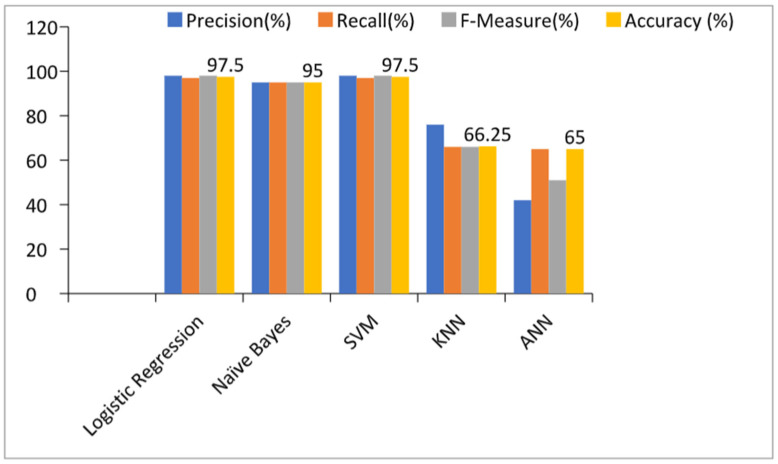
Results of the prediction models with all features. SVM: Support Vector Machine; KNN: K-Nearest Neighbors; ANN: Artificial Neural Network.

Table 3 and Figure 3 show the performance of the prediction models by considering all features or, in other words, without applying any feature selection technique. From the table and graph, we can see that the accuracies of the Logistic Regression, Naïve Bayes, SVM, KNN, and ANN-based prediction models with all features were 97.5%, 95%, 97.5%, 66.25%, and 65% respectively.

These also show that the accuracy of the Logistic Regression and SVM algorithm-based prediction models were highest i.e., 97.5%. The ANN-based prediction model achieved the lowest accuracy in the detection of kidney diseases. The performances of Logistic Regression and SVM were the same and can be used interchangeably for the detection of kidney diseases in the early stage. We can also see that the precision, recall, and F-measure values were the highest for the Logistic Regression and SVM-based prediction models.

### 5.3. Prediction Models with RFE Feature Selection Technique

Recursive Feature Elimination (RFE) is a feature selection algorithm of the wrapper type. It internally uses filter-based techniques; however, it is different to the filter approach. It has two important configuration options: a. it specifies the number of features to be selected, and b. it sets the machine learning algorithm in choosing the features. In the first case, it searches a subset of features by considering all features present in the training dataset and removes the features until the required number of features remains. In the second case, it uses a machine learning algorithm and ranks the features by their importance. It discards the least important features and repeats the model fitting process. The whole process is repeated until the mentioned number of features remains.

**Figure 4:**
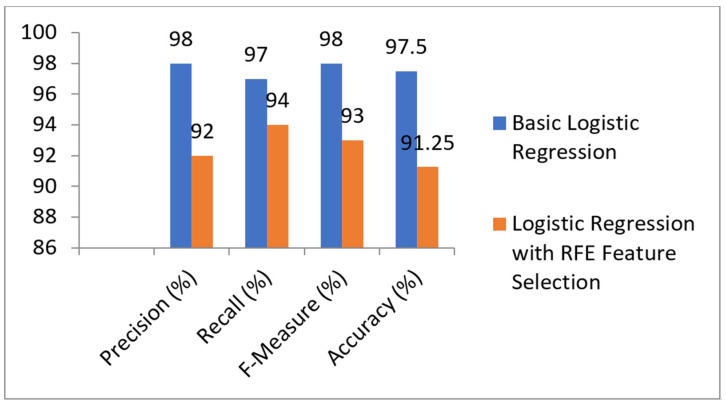
Comparison of LR Models with and without RFE feature selection. RFE: Recursive Feature Selection.

Table 4 and Figure 4 show the results of the prediction models built with basic logistic regression and with the RFE feature selection technique. From the table and graph, we can see that it achieved 97.5% accuracy without feature selection and 91.25% accuracy with RFE feature selection. It was also observed that the values of precision, recall, and F-measure were better without the RFE feature selection technique. Therefore, we conclude that the accuracy of the basic logistic model is higher than that with the RFE feature selection technique. Table 5 shows the results of the prediction models built with basic SVM and with the RFE feature selection technique.

From this, we can see that it achieved 97.5% accuracy without feature selection and 96.25% accuracy with RFE feature selection. It was also observed that the values of precision, recall, and F-measure were also better without the RFE feature selection technique. Therefore, we conclude that the accuracy of the basic SVM model is higher than that with the RFE feature selection technique.

### 5.4. Performance of Prediction Models with Chi-Square Feature Selection

In this subsection, from Table 3, we found that the accuracy of the Logistic Regression-based model was highest among the other built models in the detection of kidney disease. As we know, the feature selection technique may improve the performance of the model. In this section, we applied the Chi-Squared (chi2) statistical test to select the K-best features from the kidney disease-prediction dataset.

The Chi-Square feature selection method checks the relationship between input features and the target class. In this test, Chi-Square is determined among each input feature and the target class. It provides the required number of features with the best Chi-Square scores. It selects only those input features that have the maximum Chi-Square values. The scikit-learn library provides the SelectKBest class that is used to select a specific number of features in a suite of different statistical tests. Table 6 shows the scores of various features. It shows that the Wbcc, Bgr, Bu, Sc, Pcv, Al, Hemo, Age, Su, Htn, Dm, and Bp features have high scores in comparison with the other features.

**Figure 5:**
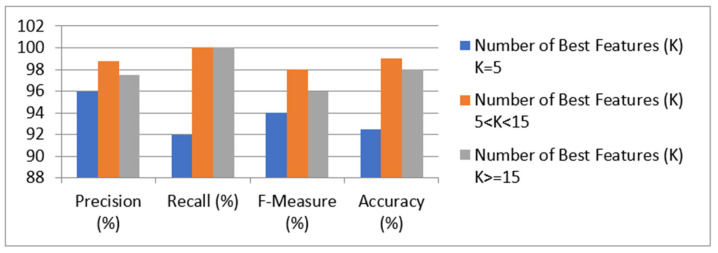
Results of the LR prediction model with Chi-Square feature selection.

Table 7 and Figure 5 show the performance of the LR prediction model with the Chi-Square feature-selection technique.

We evaluated the technique using a variety of best features. It was discovered that, when the k-values ranged from 6 to 14, the model provided the best precision, recall, f-measure, and accuracy, i.e., 100 percent, 98 percent, 99 percent, and 98.75 percent, respectively. When k = 5 or fewer features are used, the model had the lowest accuracy. The table also shows that, when more than 15 features were used, the model’s performance suffered. As a result, it can be concluded that the model with more than 5 and less than 15 features provided the highest accuracy in detecting kidney disease. The performance of the SVM prediction model with the Chi-Square feature-selection technique is shown in Table 8.

We evaluated the technique with different numbers of best features. It was found that the model achieved the best precision, recall, f-measure, and accuracy when the k-values were greater than 15, i.e., 100%, 96%, 98%, and 97.5%, respectively. The model gave the lowest accuracy when K = 5 or a smaller number of features was taken. From the table, we also see that the performance of the model decreased whenever fewer than 15 features were taken. Therefore, it can be concluded that the accuracy of the SVM model did not increase by applying the Chi-Square test.

### 5.5. Comparison of Models with and without Feature Selection Technique

**Figure 6:**
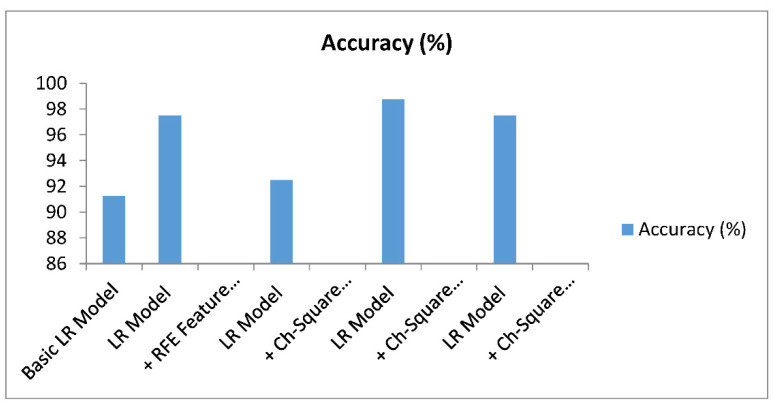
Results of the models with and without feature selection. LR: Logistic Regression; REF: Recursive Feature Elimination.

From all of the results, it can be seen that the accuracy of the Logistic Regression model with the Chi-Square feature selection techniques was the best in the detection of kidney disease. This result was the best among the other approaches in the detection of kidney disease. Table 9 shows the results of various combinations of LR models and Figure 6 graphically compares the accuracy of the different models.

The accuracies of the basic LR model, LR model with RFE feature selection, LR model with Chi-Square feature selection (K = 5), LR model with Chi-Square feature selection (5K14), and LR model with Chi-Square feature selection (K > 14) were 91.25 percent, 97.5 percent, 92.5 percent, 98.75 percent, and 97.5 percent, respectively, as shown in Table 9. This demonstrates that the Chi-Square method outperformed the RFE feature method in terms of accuracy. It is also worth noting that the model produced good results, with 5 to 15 of the best features out of a total of 24. In summary, we achieved 98.5 percent accuracy in detecting kidney disease. In comparison to existing approaches, this has the highest accuracy.

As a result of the random forest algorithm, 250 positive samples (TP) and 150 negative samples (TN) were correctly identified as positive. Positive (TP) samples were scored at 94.74 percent by the SVM, KNN, and Decision Tree algorithms with an error (TN) of 5.26 percent each, and 97.37 percent by the SVM, KNN, and Decision Tree algorithms with an error (TN) of 1.32 percent each. Table 6 shows the results of the four classifiers that were used. The random forest method outperformed the other classifiers on all metrics, including accuracy, precision, recall, and F1-score. The decision tree algorithm came in second, with accuracy, precision, recall, and F1-score values of 99.17 percent, 100 percent, 98.68 percent, and 99.34 percent, respectively. As a result, the KNN algorithm achieved 98.33 percent accuracy, precision, recall, and an F1-score of 98.67 percent. The final SVM accuracy, precision, recall, and F1-score were 96 percent, 92 percent, 93 percent, and 97 percent, respectively.

## 6. Conclusions and Future Work

In this paper, we developed many prediction models by using different machine learning algorithms and feature-selection techniques. We used a dataset that contained a large set of healthy and unhealthy patients with kidney disease. We used LR, SVM, and many other classifiers to develop various prediction models. We exercised the prediction models with Recursive Feature Elimination (RFE) and Chi-Square test feature selection techniques. From the results, it was shown that the accuracy of the Logistic Regression model with the Chi-Square feature selection technique achieved the best result in the detection of kidney disease. This result was the best among other approaches in the detection of kidney disease. It was also observed that the model achieved good results with 5 to 15 best features among 24 features. It was also found that the Wbcc, Bgr, Bu, Sc, Pcv, Al, Hemo, Age, Su, Htn, Dm, and Bp features had more significance in the detection of kidney diseases. In the future, we will develop a hybrid approach for improving disease detection accuracy before actual disease arises in humans.

## Figures and Tables

**Table 1:** Summary of related work.

Sr. No.	Author	Year	Machine Learning Algorithms and Accuracy (%)
1.	A. J. Aljaaf et al. [1]	2018	Naïve Bayes: 83.4%, J48: 86.23%
2.	N. Borisagar, D. Barad, and P. Raval [5]	2017	ANN: 99.5
3.	B. Boukenze, A. Haqiq, and H. Mousannif [6]	2018	SVM: 63.5%, LR: 64.0, C4.5: 63%, KNN: 55.15%
4.	H. Polat, H. D. Mehr and A. Cetin [7]	2019	SVM: 97.5%
5.	P. Panwong and N. Iam-On [8]	2016	KNN: 86.32%, naïve Bayes: 60.46%, ANN: 83.24%, RF: 86.60%, J48: 79.52%
6.	Makino et al. [11]	2019	KNN, Naïve Bayes + LDA + random subspace + Tree-based decision: 94%
7.	Ren et al. [12]	2019	SVM + ReliefF: 92.7%
8.	Ma F. et al. [13]	2019	Fisher discriminatory analysis and SVM: 96.7%
9.	Almansour and colleagues [14]	2020	KNN and SVM: 99%
10.	J. Qin and colleagues [15]	2019	SVM, KNN, and naïve Bayes decision tree: 99.7%
11.	Z. Segal and colleagues [16]	2019	SVM, KNN, and decision tree: 99.1%
12.	Khamparia et al. [17]	2020	Logistic regression, KNN, SVM, random forest, naive Bayes, and ANN: 99.7%
13.	Ebiaredoh-Mienye Sarah A. et al. [18]	2017	SVM 98.5%
14.	Zhiyong Pang et al. [19]	2020	Softmax regression 98%
15.	Tabassum, Mamatha et al. [23]	2017	DT: 85%, RF: 85%
16.	K. R. A. Padmanaban and G. Parthiban [24]	2016	DT: 91%, naïve Bayes: 86%
17.	Sahil Sharma, Vinod Sharma, and Atul Sharma [25]	2018	ANN: 80.4%, RF: 78.6%
18.	Pratibha Devishri [26]	2019	ANN: 86.40%, SVM: 77.12%
19.	Sujata Drall, G. Singh Drall, S. Singh, Bharat Naib [27]	2018	Naïve Bayes: 94.8%, KNN: 93.75%, SVM: 96.55%

LR: Logistic Regression; KNN: k-Nearest Neighbors; SVM: Support Vector Machines; CART: Classification and Regression Trees; ANN: Artificial Neural Networks; LDA: Linear Discriminant Analysis; DT: Decision Tree; RF: Random Forest.

**Table 2:** Details of the various kidney disease-related attributes.

Name	Feature	Description
Age	Age	Patient’s age
Blood pressure	Bp	Blood pressure of the patient
Sugar level	Su	Sugar level of the patient
Bacteria	Ba	Presence of bacteria in the blood
Ratio of the density of urine	Sg	Ratio of the density of urine
Albumin level in the blood	Al	Ratio of the albumin level in the blood
Pedal edema	Pe	Does the patient have pedal edema or not
Red blood cells	Rbc	Patients’ red blood cell counts
Patient class	Class	Does the patient have kidney disease or not
Pus cell clumps	Pcc	Presence of pus cell clumps in the blood
Anemia	Ane	Does the patient have anemia or not
Red blood cell count	Rc	Red blood cell count of the patient
Hypertension	Htn	Does the patient have hypertension on not
Serum creatinine	Sc	Serum creatinine level in the blood
Diabetes mellitus	Dm	Does the patient have diabetes or not
Blood urea	Bu	Blood urea level of the patient
Blood glucose	Bgr	Blood glucose random count
Sodium	Sod	Sodium level in the blood
White blood cell count	Wc	White blood cell count of the patient
Hemoglobin	Hemo	Hemoglobin level in the blood
Packed cell volume	Pcv	Packed cell volume in the blood
Pus cell	Pc	pus cell count of patient
Potassium	Pot	Potassium level in the blood
Appetite	Appet	Patient’s appetite
Coronary artery disease	Cad	Does the patient have coronary artery disease or not

**Table 3:** Results of the prediction models with all features.

Machine Learning	Precision	Recall	F-Measure	Accuracy
Algorithms	(%)	(%)	(%)	(%)
Logistic regression	98	97	98	97.5
Naïve Bayes	95	95	95	95
Support Vector Machines	98	97	98	97.5
k-Nearest Neighbors	76	66	66	66.25
Artificial Neural Networks	42	65	51	65

**Table 4:** Results of the LR model with RFE feature selection technique.

Performance Measure	Basic Logistic Regression	Logistic Regression with RFE Feature Selection
Precision (%)	98	92
Recall (%)	97	94
F-Measure (%)	98	93
Accuracy (%)	97.5	91.25

RFE: Recursive Feature Selection.

**Table 5:** Results of the SVM model with the RFE feature selection technique.

Performance Measure	Basic SVM	SVM with RFE Feature Selection
Precision (%)	98	98
Recall (%)	97	96
F-Measure (%)	98	97
Accuracy (%)	97.5	96.25

SVM: Support Vector Machine; RFE: Recursive Feature Elimination.

**Table 6:** Features and their scores by the Chi-Square test.

Features	Score
Wbcc	12,733.73
Bgr	2428.328
Bu	2336.005
Sc	354.4105
Pcv	324.7065
Al	228.1047
Hemo	125.0657
Age	113.4602
Su	100.95
Htn	86.29181
Dm	82.2
Bp	80.02432
Pe	45.10802
Ane	35.6116
Sod	28.7933
Pcc	24.07546
Rbcc	20.848
Cad	19.93604
Pc	14.16913
Ba	12.58705
Appet	12.58703
Rbc	9.416036
Pot	4.071145
Sg	0.005035

Wbcc: White Blood Cell Count; brg: Blood Glucose Random; Bu: Blood Urea; Sc: Serum Creatinine; Pcv: Packed Cell Volume; Al: Albumin; Hemo: Hemoglobin; Su: Sugar; Htn: Hypertension; Dm: Diabetes Mellitus; Bp: Blood Pressure; Pe: Pedal edema; Ane: Anemia; Sod: Sodium; Pcc: Pus cell clumps; Rbcc: Red blood cells count; Cad: Coronary artery disease; Pc: Pus cell; Ba: Bacteria; Appet: Appetite; Rbc: Red blood cells; Pot: Potassium; Sg: Ratio of the density of urine.

**Table 7:** Results of the LR prediction model with Chi-Square feature selection.

Performance Measure	Number of Features (K)	Best K > = 15
	K = 5 5 < K < 15	
Precision (%)	96 100	100
Recall (%)	92 98	96
F-Measure (%)	94 99	98
Accuracy (%)	92.5 98.75	97.5

**Table 8:** Comparative analysis of existing models on a dataset of 400 patients each with 24 attributes [2,27].

Method	Accuracy	Recall	Precision	F-Measure
Logistic regression [28]	91.8	1	0.98	0.98
KNN [29]	92.7	0.88	0.98	0.92
Naïve Bayes [30]	95.21%	0.92	1.00	0.94
SVM [31]	92.32	0.87	0.96	0.93
Decision tree [32]	93.45	0.95	1.00	0.96
Proposed method [33]	97.54	0.99	1.00	1.0

KNN: k-nearest neighbors algorithm; SVM: support vector machines.

**Table 9:** Prediction models with and without various feature-selection techniques.

Prediction Model	Accuracy (%)
Basic LR model	91.25
LR model + RFE feature selection	97.5
LR model + Chi-Square feature selection (K = 5)	92.5
LR model + Chi-Square feature selection (5 < K < 14)	98.75
LR model + Chi-Square feature selection (K > 14)	97.5

## Data Availability

Data is available on reasonable request.
